# Compliance of a cobalt chromium coronary stent alloy – the COVIS trial

**DOI:** 10.1186/1468-6708-6-17

**Published:** 2005-10-28

**Authors:** Jens Hagemeister, Frank M Baer, Robert HG Schwinger, Hans W Höpp

**Affiliations:** 1 Department of Medicine III, University of Cologne, Kerpener Str. 62, 50924 Cologne, Germany

## Abstract

**Background:**

Cobalt chromium coronary stents are increasingly being used in percutaneous coronary interventions. There are, however, no reliable data about the characteristics of unfolding and visibility of this stent alloy *in vivo*. The aim of this study is to compare cobalt chromium coronary stents with conventional stainless steel stents using intracoronary ultrasound.

**Methods:**

Twenty *de novo *native coronary stenoses ≤ 20 mm in length (target vessel reference diameter ≥ 2.5 and ≤ 4.0 mm) received under sequential intracoronary ultrasound either a cobalt chromium stent (Multi-Link Vision^®^; n = 10) or a stainless steel stent (Multi-Link Zeta^®^; n = 10).

**Results:**

For optimal unfolding, the cobalt chromium stent requires a higher balloon deployment pressure (13.90 ± 2.03 atm) than the stainless steel stent (11.50 ± 2.12 atm). Furthermore, the achieved target vessel diameter of the cobalt chromium stent (Visibility-Index QCA/IVUS Multi-Link Vision^®^1.13 / Multi-Link Zeta^® ^1.04) is more easily overrated by Quantitative Coronary Analysis.

**Conclusion:**

These data indicate that stent material-specific recommendations for optimal implantation pressure and different stent material with an equal design should both be considered in interpreting QCA-analysis.

## Background

With the addition of coronary stents to percutaneous coronary intervention (PCI), the incidence of re-stenosis has been significantly reduced. Unfortunately, re-stenosis rates still range from 16% to 32 %[1]. Efforts to reduce re-stenosis include coating of conventional stents and use of alternative materials and design. Drug Eluting Stents (DES) are already established in clinical practice[2], whereas little data are available with respect to innovative stent material.

Cobalt chromium represents a more biocompatible material that is being increasingly used in coronary stents (Guidant Multi-Link Vision^®^/Guidant Corporation, Driver-Stent^®^/Medtronic, Costar-Stent^®^/Biotronic). In comparison with stainless steel, cobalt chromium has a higher radial strength and radiopacity for similar electronegativity. This allows for the production of thinner struts with a similar radiological visibility[3].

Although, results of two cobalt chromium registries[3,4] are already published, there are no data describing the basic characteristics of unfolding of a cobalt chromium stent. This information would be important to know in developing clinical recommendations for different alloys, since optimal inflation and complete adherence to vessel wall are key factors affecting the incidence of re-stenosis.

We investigated the balloon deployment pressure-related behaviour of a cobalt chromium stent (Multi-Link Vision^®^), comparing it to a similarly designed conventional stainless steel stent (Multi-Link Zeta^®^) using intravascular ultrasound. We also evaluated radiological visibility of both stents and the influence of radiological visibility on QCA-analysis.

## Methods

Eighteen consecutive patients (14 men, 4 women, mean age 61 ± 9 years) with twenty single, *de novo *native coronary stenoses ≤ 20 mm in length were blindly randomised to either a Multi-Link Vision^® ^cobalt chromium stent (n = 10) or to a Multi-Link Zeta^® ^stainless steel stent (n = 10). Additional criteria for inclusion were age ≥ 18 years, clinical angina and/or a positive functional study and a target vessel reference diameter ≥ 2.5 mm and ≤ 4 mm.

Patients were excluded from the study if they presented with cardiogenic shock, acute coronary syndrome, intracoronary thrombus, vessel occlusion, target lesions in the left main artery, ostial or bifurcational stenosis, calcification of ≥ 180° of vessel circumference by intravascular ultrasound (IVUS), diabetes and/or known hypersensitivity to aspirin and clopidogrel. The study was approved by the ethics committee of the University of Cologne and all patients signed written informed consent before participating.

Percutaneous coronary intervention was performed in accordance with standard clinical procedures, and stent implantation without predilatation ("primary stenting") was encouraged. Pretreatment included an oral clopidogrel loading dose of 300 mg and aspirin 500 mg the day before intervention. Peri-interventional, weight-adapted heparin was given intravenously with consecutive control of activated clotting time. Implantation was performed with a primary balloon deployment pressure of 10 atmospheres (atm). If IVUS target criteria were not reached, a standardised further inflation with 13 atm and possibly 16 atm including additional IVUS control, followed. Post-procedural an oral antiplatelet therapy with clopidogrel 75 mg/day for at least 4 weeks and aspirin 100 mg/day as standard medication was obligatory.

Intravascular ultrasound imaging was performed after administering 0.2 mg of intracoronary nitroglycerin using a 30 MHz transducer within a 3.2 Fr imaging sheath (SCIMED/BSC, Maple Grove, Minnesota) and automatic transducer pullback of 0.5 mm/s. The distal and proximal reference segment was within 3 – 5 mm of the lesion or stent without a relevant stenosis (<20%). Quantitative Coronary Analysis (QCA, Pie Medical Imaging) was done for proximal and distal reference diameter (RD), minimal lumen diameter (MLD), diameter of stenosis and acute lumen gain.

The primary endpoint was balloon deployment pressure once IVUS criteria were reached. IVUS target criteria were similar to preceding studies[5], with a minimal lumen area (MLA) after stenting > 90% (for reference lumen area (RLA) ≤ 9.0 mm^2^), > 80% (for RLA > 9.0 mm^2^), a MLA > 90% of proximal RLA at the proximal end of the stent and a complete adherence to vessel wall. A secondary endpoint was angiographic visibility as generated by minimal lumen diameter in QCA and IVUS-analysis, respectively.

Statistical analysis was performed with SPSS, version 12.0 (SPSS Inc., 2003). Continuous variables are expressed as mean ± SD.

## Results

Clinical data were comparable for both groups, except for the number of smokers (Vision^® ^n = 7 / Zeta^® ^n = 3), and were comparable to prior stent studies. Twenty five stents (13 ML Vision^®^, 12 ML Zeta^®^) were implanted with a 100% procedural success. An intraprocedural dissection in 3 patients required implantation of additional stents. In case of stent overlap, the same stent type was used.

The angiographic mean reference vessel diameter was 3.02 ± 0.40 mm (ML Vision^® ^3.04 ± 0.34 mm / ML Zeta^® ^2.99 ± 0.47 mm). Mean minimum lumen diameter was 0.70 ± 0.32 mm, which corresponds to a mean diameter stenosis of 76.5 ± 9.8% (Table 1).

**Table 1:** Baseline Lesion Characteristics (QCA).

	**ML Vision (n = 10)**	**ML Zeta (n = 10)**
Preprocedure reference vessel diameter (mm)	3.04 ± 0.34	2.99 ± 0.47
Preprocedure MLD (mm)	0.77 ± 0.32	0.63 ± 0.32
Preprocedure diameter stenosis (%)	73.50 ± 10.30	79.60 ± 8.80
Target vessel		
Left anterior descending	20%	50%
Circumflex	20%	20%
Right coronary artery	60%	30%
Postprocedure MLD (mm)	3.34 ± 0.34	3.31 ± 0.48
Postprocedure diameter stenosis (%)	6.00 ± 1.80	5.10 ± 3.80

Pre-dilatation was necessary in one case, in which a 1.5 × 20 mm balloon was used and dilatation pressure was 8 atm.

**Figure 1:**
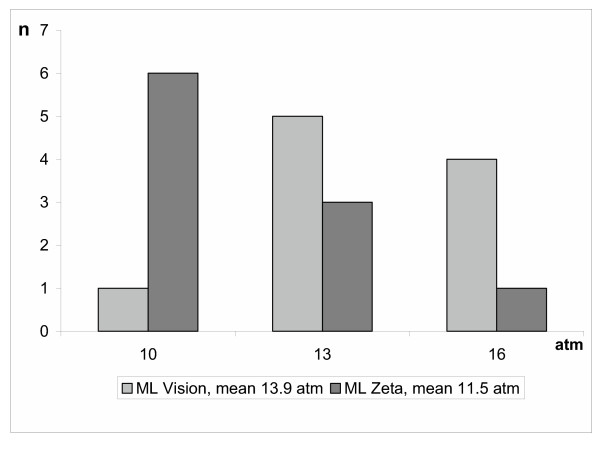
**Ballon pressure by IVUS-criteria**: balloon deployment pressure when IVUS criteria were reached for Multi-Link Vision^® ^and Multi-Link Zeta^® ^(n = number of lesions).

Mean balloon deployment pressure when IVUS criteria were reached was 13.90 ± 2.03 atm for Multi-Link Vision^® ^and 11.50 ± 2.12 atm for Multi-Link Zeta^® ^(Figure 1).

A mean post-procedural QCA-MLD of 3.32 ± 0.41 mm and a mean MLD of 3.07 ± 0.41 mm by intravascular ultrasound generated a mean Visibility-Index of 1.08. For Multi-Link Vision^® ^(3.34 ± 0.34 mm [QCA] vs. 2.95 ± 0.17 mm [IVUS]), mean Visibility-Index was 1.13. The mean Visibility-Index for Multi-Link Zeta^® ^(3.31 ± 0.48 mm [QCA] vs. 3.19 ± 0.55 mm [IVUS]) was 1.04 (Table 2).

**Table 2:** Visibility-Index: generated from QCA- and IVUS-data.

	**All**	**ML Vision**	**ML Zeta**
Postprocedure MLD QCA (mm)	3.32 ± 0.41	3.34 ± 0.34	3.31 ± 0.48
Postprocedure MLD IVUS (mm)	3.07 ± 0.41	2.95 ± 0.17	3.19 ± 0.55
Visibility [QCA/IVUS]	1.08	1.13	1.04

## Conclusion

Different material properties of alloys used in a specific stent design represent a key consideration in clinical practice that should be taken into account, particularly with regard to balloon deployment pressure. Furthermore, the achieved post-procedure lumen diameter as measured by QCA is more easily overestimated for the cobalt chromium stent than for the stainless steel stent due to material properties.

Based on these results, further investigation of material-specific changes in stent unfolding is necessary to guide and optimize the clinical implantation practice.

## Limitations

The number of twenty *de novo *native coronary stenoses is small, but relevant preliminary differences were found that should be further explored in future investigations.

Three standardised balloon deployment pressures (10, 13, 16 atm) were chosen because every change between balloon and IVUS-catheter increases the risk of vascular damage. Thus, a continuous, incremental escalation of balloon deployment pressure is not practical *in vivo*.
